# A Pulpit Defence of the Stage

**Published:** 1893-03

**Authors:** R. H. Conwell


### A PULPIT DEFENCE OF THE STAGE.

I am opposed to the theatre because it comprehends so much that is offensive to good taste and good morals. Having been acquainted in years past with the stage and some of its most prominent exponents, I can say with equal frankness that there are some actors and some plays and operas which, by themselves, are true in character and ennobling in their influence. There are generous, clean, and honorable gentlemen and ladies on the stage, whose upright example and unspotted lives serve to bolster up a bad lot of questionable hangers on and vile impersonators. Actors and managers will agree with me in this statement. The grand productions of the great authors and great actors serve often to make other places, authors and actors respected and patronized which do the community great harm. What to do about it I do not see. If the excellent plays and best actors could be distinctly separated from the inartistic and deleterious, so that patrons could always distinguish beforehand what would be elevating and moral, then the pulpit and the stage could and ought to join hands. Grand scenery, delicious music, perfect elocution and the highest cultivation of voice and gesture are mighty accessories in teaching truth, charity, heroism and religion. They all ought to be used to purify and inspire humanity with noble motives. But when there is no line drawn and the same presents Jefferson and the "Devil's Auction," or Shakspeare and the "Black Crook," the bad drags down the good and encourages the pure to listen to impure things. I believe the time is to come when we will have theatres and music halls in which only pure music, excellent acting, moral plays and the best classics will be heard or seen. But first how to secure that desirable end is a problem to which all good men on the stage and off may profitably devote most serious study. Cannot the wheat be separated from the chaff, the noble from the ignoble, the high from the low, the true from the false, and secure a safe presentation of the highest music, the grandest dramas and the purest fun? Think of it.—*Rev. R. H. Conwell, in Detroit Free Press.*

